# Healthcare Practice Post COVID-19 Impacts: Will 21st Century Pharmacists Become Global, Agile, Collaborative and Curated?

**DOI:** 10.3390/pharmacy13060162

**Published:** 2025-11-03

**Authors:** Maree Donna Simpson, Jaimy Jose, Jennifer L. Cox

**Affiliations:** School of Dentistry and Medical Science, Charles Sturt University, Orange, NSW 2800, Australia; masimpson@csu.edu.au (M.D.S.); jjose@csu.edu.au (J.J.)

**Keywords:** health professional education, curation, collaboration, professional practice, natural disasters, pharmacy

## Abstract

“*Those that fail to learn from history are doomed to repeat it.*” Winston Churchill. In recent times, globally, approximately three pandemics and thousands of natural disasters and political upheavals have been recorded. In most cases, tens to hundreds of thousands of people have died as a result, whether from droughts, famines, floods, earthquakes, tsunamis, wildfires, landslides, cyclones, typhoons, hurricanes, extreme heat, emerging or resurgent diseases or longer-term issues such as sustainability, climate change and/or global warming. Whilst many accommodations may have been made to cope with these, we propose that pharmacy education and professional practice benefit from learning from the past, from collaboration globally to manage the hectic and uncertain times that result from these disruptions and from curation and evaluation of these initiatives for ongoing and/or future use.

## 1. Introduction

Education for the 21st century reflects practice, technological and structural changes from earlier times. For example, in the early-mid 20th century, community pharmacists were often practising as single practitioners seeing individual patients in a discrete practice site addressing acute illnesses or injuries. However, hospital pharmacists practised as part of a pharmacy team with responsibilities to out-patients, in-patients by health needs. Coming into the 21st century, patients now seek ongoing care for chronic health conditions, from a multidisciplinary team in broader community settings concerned with population health [1,2], and hospital pharmacists more frequently as a member of multidisciplinary team.

Education for the 21st century likewise needs to reflect positive and negative impacts on health, such as significant drug discoveries, technological change and development during the 20th century and through to current times, emerging and re-emerging diseases [3], and obesogenic lifestyles and information seeking from online sources, colloquially identified as “Dr. Google” [4]. Health education also benefits from considering the impacts of generational change [5], smart phones [6], artificial intelligence [7], 3D printers for medicines [8], smart bottles [9], and virtual reality [10], keeping current with new and emerging technology.

### Observations on the Development of Professionals and Practice: Insights from History

From the early days of humanity, there were people with different skills who offered different services to their communities. Those people, under a variety of titles, were responsible for caring for the sick and the injured. In time, these roles were characterised as professions with expectations of each profession to society and the reciprocity of the community to the professions. This relationship became known as a social contract.

Over time, many different groups of people caring for the sick and injured were recognised as professionals, such as nurses, dentists, pharmacists (apothecary/chemist), physiotherapists, occupational therapists, and emerging groups such as exercise physiologists. Along with this development has come the expectation of some standardisation and comparability within health professional groups. This led to incremental change in the preparation for practice from apprenticeship models to apprenticeship plus some formal foundational knowledge, to formal academic qualifications which may lead to practice or to an intern training period, with a final review or assessment leading to registration as a fully fledged health professional, such as a pharmacist.

Pharmacy curricula are based on foundational sciences such as physiology, clinical knowledge and skills relevant to the pharmacy profession, and social sciences and skills, particularly the social determinants of health. The social determinants of health are important as they are reported, for example, to contribute to or account for 25 to 60% of deaths in the United States in any given year [11]. Conceptualisations of the social determinants may vary a little but essentially there are factors at two levels. The first level is at the individual level and includes education, employment status, housing and food availability and safety; the second level is at the community level and includes factors such as air pollution levels, personal security and safety and housing quality [11].

So, health is conceptualised as more than an absence of disease or injury but rather as the interplay of biological factors such as genetic inheritance; access to health services not only for treatment but also for screening and monitoring for health conditions; environmental factors such as climate change [12,13]; natural disasters such as floods [14], wildfires [15], tsunami [16] or earthquakes [17]; and the individual’s lifestyle choices and practices such as diet, exercise, and stress management. This highlights the reality that there are challenges to health in the 21st century, with six basic principles identified. These are (a) disease prevention and health promotion, (b) reducing inequalities in health, (c) cooperation between different sectors of society, (d) active community engagement, (e) primary healthcare and (f) international cooperation [18].

To date, the following changes have been observed—more years of education, increasing training and professional development for expanded scope activities, a higher registerable qualification such as Doctor of Pharmacy (Pharm. D.), more use of online learning, more preparation to participate in telehealth, healthcare robots [19], use of high- fidelity simulations to learn and demonstrate professional skills. Of concern, emerging digital health issues have been identified: computer vision syndrome [19]; text neck or tech neck [20]; digital dementia [21,22]; internet “addiction” or “always on culture” [23,24,25]; and selfie elbow [26].

Pharmacy curricula are informed by foundational knowledge, clinical reasoning, decision-making strategies, ethical behavioural expectations, effective communication skills, anticipations of empathy and caring, and development of professionalism. Nonetheless, curricula, even within the same nation, region or city, may utilise different learning modalities to achieve these objectives. Selected modalities or influences will be discussed in greater detail below.

In the twentieth century and in the twenty-first century, communities around the globe have been affected by infectious diseases, such as the 1918 pandemic influenza, 2009 H1N1 influenza, and more locally such as Ebola, Middle East Respiratory Syndrome (MERS) and Zika virus [27]. In late 2019, a novel coronavirus (SARS Cov-2) was identified, and the condition which resulted from infection was and is referred to as COVID-19. The World Health Organisation declared a global outbreak, which resulted in a pandemic on 11 March 2020. Some commentators declared COVID-19 to be a “once in a lifetime pandemic” given the level of morbidity and mortality inherent in the condition. Initially, there were no recognised vaccines produced, nor approved medications to treat the condition, with over 2.4 million deaths reported by late February 2021 [27]. In an attempt to contain COVID-19, local, regional and global lockdowns resulted; however, during the lockdowns, most community and hospital pharmacies remained accessible to provide necessary medications for acute and chronic conditions; support quality use of medicines especially antimicrobial stewardship; manage medication shortages and acceptable alternatives, hand sanitiser products, antiseptic and disinfectant products, personal protective equipment such as masks and gloves, and consumer products such as tissues, toilet paper and paper towels. Once vaccines became available, pharmacists were identified in many nations as accessible and effective vaccinators [28,29], assisting in the rollouts as supplies became more available, and in mass vaccinations of health professionals [30]. Subsequent to this activity, pharmacists in developed countries, and in some low-resource nations, have seen significant and often substantial expansion of the scope of practice into, for example, prescribing [31], therapeutic substitution [32], and public health roles [33].

For many pharmacists, the net effect was to be recognised as frontline health professionals and often valued for their commitment [34]. Indeed, Hennekam and colleagues [34] characterised them and other non-physician healthcare workers as being viewed by the public as “heroes” rather than “invisible” according to previous perspectives. However, with medication shortages and other availability challenges, not all pharmacists received recognition as heroes [35], with some in the United States of America and globally being abused and on occasion assaulted, reflecting frustration with the shortage of prescription medicines and consumables such as tissues, paper towels, hand sanitiser and toilet paper.

Whilst natural disasters such as floods, droughts, wildfires, and earthquakes may cause changes to practice in healthcare workers, the effects are more localised than a global pandemic. Wars and political upheavals vary in their impact based on scale, such that a World War would likely affect most nations, whilst a political upheaval more often affects a single nation or two divided nations.

Even in a global pandemic such as COVID-19, different outcomes and lessons have been observed and reported. In the United Kingdom, for example, healthcare workers were reported to have different behaviours and concerns, such as fear of infection in themselves, concern for their families’ health and safety, some experience of stigma, and the desire for more support from their organisations, colleagues, governments and regulatory bodies.

## 2. Professionalism and Changing Needs and Practices

Professionalism is generally considered to reflect personal integrity and trustworthiness, plus altruism, humanistic values, accountability, ethical practices, clinical competence and culturally safe communication [36]. Professionalism is a strongly desired characteristic for health professionals and health professional students. But what characterises “professionalism” has changed across the 20th century and into the 21st century, reflective of significant global changes. The agents of change are many but include emerging and re-emerging infectious diseases, climate change, ageing populations, digital technology, AI, environmental hazards such as micro-plastics, different lifestyle choices, rural-to-urban population shifts, and uncertain times [36].

However, are students taught professionalism consistent with 21st century values and lifestyles? Are the traditional humanistic values even more important in this time, when AI guides surgical robots, provides diagnoses of skin cancers, manages autonomous vehicles, or are they less important? Further, pharmacists are not protected from working with robots, nor from associated ethical challenges. Table 1 highlights emerging health support technologies, such as dispensing robots, which are currently utilised, along with potentially autonomous mobility scooters and personal care and social robots, which may be widely stocked in pharmacies in the future. We argue that humanistic values are even more important but in need of additions to address the vastly different world in which students will practice as future pharmacy practitioners.

## 3. Professional Identity Development

Developing an effective professional identity requires intending health professionals to internalise and demonstrate the behavioural norms, morals, ethics, standards and competencies of their intended professional community. So that one comes, over time, to “walk the walk, and talk the talk” or to think, act and feel like a member of the profession they seek to join [43].

Curricula usually incorporate several activities that demonstrate successful professional identity, and while some students absorb and incorporate the relevant information and communication style, and develop the necessary skills seemingly effortlessly, others find the development more challenging. COVID-19 had a number of impacts, some strengthening perceptions of professional identity as student volunteers joined the “surge” workforce, and others that impacted negatively, causing students to feel unsettled in their future profession and their current course. Similarly, natural disasters, pandemics and wars also impact professional identity development [44].

There are many factors that impact the development of a robust professional identity; however, as Trede asserts [45] though every professional has “a” professional identity, some professionals may be unable to articulate their future professions’ professional values and delineate them from personal beliefs or perceptions unless well prepared for their future work role in authentic sites during experiential learning. Trede proposes that students benefit from the application of disciplinary knowledge and technical skills, but as importantly, experiential learning in authentic sites helps students develop self-leadership skills and capacities such as communicating effectively with others, working collaboratively in a team, and learning workplace argot and culture [45].

However, lessons from the global pandemic and the associated changes in healthcare, such as increased remote health practices, raise questions about the sustainability of experiential learning as a predominant educational intervention for professional identity development. Table 2 displays examples of experiential learning and some alternatives utilised during COVID-19.

## 4. Discussion

### 4.1. The Emerging Role of Technology (AI, Diagnosis, Surgery, 3D Printing Medicines)

COVID-19 has not only impacted the health and behaviours of people globally, it has also impacted healthcare and the modes of offering healthcare. With initial uncertainty about transmission and public health responses such as lockdowns, digital health was viewed as a potential contributor to management and solutions with adaptations such as telehealth and electronic health records [57]. However, while any innovation has potential benefits, it also comes with some challenges, such as patient privacy and rights, with equity and security issues rapidly evolving societal healthcare needs [57].

Our students benefit from developing the knowledge arising from this global pandemic, building skills in the safe and effective use of AI, telehealth etiquette and technology operation for the security of data collected or shared and protective environmental modifications, such as screens and personal protective equipment to communicate effectively with patients, maintaining privacy and confidentiality.

### 4.2. Global Health, Coping with the Unexpected, and Sustainability

One key outcome of the global pandemic arising from COVID-19 was the necessity to think globally, to collaborate broadly, and to then act locally or regionally, consistent with needs and resource base. This was well exemplified by the global “World Café” held by health professional educators and reported in 2020 [58]. This gathering resulted from a strategy to cross geographical boundaries to reflect on common educational challenges and share ideas and solutions. Further, this “World Café” venue facilitated networking and mentorship amongst educators to engage with and support others undergoing similar experiences. This facilitated new approaches to educational goals by bringing together experienced and less experienced educators whose brainstorming and sharing led to synergies that often generated novel solutions and also lessened the feelings of isolation that responses to COVID-19, such as lockdowns or working only from home with online teaching, generated. Although not specifically identified as such, the “World Café” we would propose operated in the spirit of “One Health” as disseminated by the One Health Commission, since 21st century health issues are increasingly global and complex, blending issues such as emerging diseases and global warming, for example, and benefitting when professionals with different skills, such as researchers, practitioners and educators, seek short-term and longer-term solutions to those issues.

However, nations, national governments and global bodies benefit from considering how to allocate enough resources to educating sufficient health professionals; to developing sufficient access to healthcare whether as hospitals, community clinics, urgent care clinics, satellite hospitals or other points of care relevant to their nations; to encourage scientists, researchers and clinicians to innovate and develop projects seeking new drug developments, new modes of care such as robotic carers, new concepts of isolation, long-life shelf-stable food, sufficient personal protective equipment; and for educators to develop curricula that an unexpected pandemic, natural disaster or political upheaval cannot as easily disrupt. Perhaps incorporating insights from a Japanese concept of “Bosai”, which incorporates a wide range of activities from mitigation and prevention to post-impact management and re-development, could also be beneficial [59].

Educators, regulators, governments and key stakeholders currently have a “golden opportunity” to review the global and local needs of public health and community health to inform strategies for pharmacy students’ education going forward in the 21st century.

Some educators have sought to develop a curriculum reflective of the needs of the 21st century and the benefits of being well prepared and vigilant. For example, Huss and colleagues in 2020 [60] report that 4.4% of global greenhouse gas emissions are attributable to healthcare and identify the need for curricula to be informed by this, given the link identified between sustainable healthcare practices and the development of professional identity.

Further, Levett-Jones and colleagues incorporated a literature review to generate statements addressing sustainability knowledge, necessary nursing skills, and issues arising from climate change, followed by a Delphi study to seek consensus on these statements from 42 international experts [61]. These educators clearly identify that neither members of the public nor patients contributed to this process, yet both may be affected by the care they could receive during the next pandemic or natural disaster, war or political upheaval.

### 4.3. Technology Is Rarely Neutral

Although technology can be one of the solutions to challenges such as COVID-19, natural disasters, and unsettled times, it is important that educators, practitioners, and students remember that technology is rarely both neutral and equitable. All stakeholders benefit from being aware of the health, ethical, acceptability, affordability and policy challenges that seem inherent in technology.

Further, technology can be vulnerable to issues such as solar flares [62], software issues such as a global CrowdStrike issue recently [63], and power interruptions (short-term or longer) due to natural disasters or wars. For sustainability, back-ups or alternate sources need to be considered and readily available.

Educators, regulators, governments, and key stakeholders currently have the opportunity to review the global and local needs of public health and community health to inform health education for all health students going forward, transitioning from becoming to being health professionals in the 21st century. These needs benefit from being informed by the differential impact of COVID-19 and impacts from other future pandemics, natural disasters, wars, and political upheavals. Pharmacy registering bodies have a particular role and opportunity during trying and uncertain times and preparing for them. Registering bodies accredit pharmacy courses and register pharmacists who have met the requirements to graduate and completed activities beneficial for practice, often during an intern year post-graduation. They set standards for practice and protect their communities by maintaining registers of qualified pharmacists by upholding codes of conduct and acting proactively to maintain safe practice during trying and uncertain times. These bodies play a crucial role in supporting and maintaining the social contract of pharmacy as a profession with the communities served.

## 5. Conclusions

There is little doubt that the COVID-19 pandemic and measures to contain and manage it have affected individuals’, communities’, and nations’ lives; access to healthcare, including necessary medicines and management of chronic diseases; and access to screening and monitoring, such as mammograms. In addition, COVID-19 has changed the face of primary care and institutional care in hospitals and residential care, with healthcare workers’ practice including more administrative tasks, more telehealth, and some abuse from patients and some praise. Some pharmacists struggled with being abused and blamed for medicine shortages that were global and that they had little to no control over.

In addition, social distancing and isolation significantly affected mental health, especially feelings of anxiety, depression, and disconnection from friends, family, and their community [64]. This social isolation and disruption were also identified as having impacted social cohesion, with inappropriate behaviours such as abuse, and later criminal actions such as physical assaults, robberies, and home invasions.

On a more positive note, technology has demonstrated the capacity to spare some healthcare workers the necessary patient care, with AI and robotic carers absorbing some of that load. Most healthcare workers such as pharmacists who work in community and/or institutional settings experienced a necessary expansion of scope and decision making to address community concerns, needs and wants [27]. This impacted the education and training of pharmacists and other healthcare workers and students, as these activities transitioned from an emergency need to an expected practice.

However, the pandemic also identified a core capability that facilitates managing change in uncertain times: adaptability. This capability reflects the capacity to undertake and achieve learning goals and objectives with considered thought and strategic approaches. Zhang and colleagues [65] report on the situation in China, where students undertook to “Suspend Classes without Stopping Learning” by engaging with learning online. Their research established that adaptability, or the capacity to respond successfully to change and/or uncertain conditions, and student engagement are significantly positively correlated with positive academic emotion, and the reverse also applies [65]. They further proposed that there are three necessary components to adaptability: a cognitive domain (thoughts), a behavioural domain (actions), and an affective domain. Zhang and colleagues [65] further considered research exploring other aspects of adaptability, such as Besser and colleagues’ study [66] on students’ personality traits. This study established that the big five personality traits significantly predicted adaptability to the COVID-19 pandemic. Extraversion, openness, agreeableness, and conscientiousness were positively associated with adaptability, whilst neuroticism was negatively associated [66].

This synthesis of the results of prior work by Zhang and colleagues provides a developmental path for educators, students, and practitioners to better manage concerning and uncertain situations.

Unfortunately, in exploring the impact of COVID-19 on students’, practitioners’ and educators’ coping skills, burnout and mental health issues, some differences between nations, communities and learning approaches became apparent. Differences between pragmatic and more dramatic personality characteristics, differences between collectivist and more individualistic community characteristics [67,68,69], and differences in exposure to natural disasters [70,71,72], wars, and political upheavals [73,74] were noted. Therefore, it may require a customised approach rather than a single global approach addition, but it could benefit from sharing of information and resources where relevant [75], learning from other experiences and solutions. This re-imagining of health education, upskilling, scope modifications and practice could benefit from collaboration between the health professions, since this may include some scope expansions or sharing regulatory authorities’ roles, perhaps consolidating legal bodies with impacts on health, patients, consumers, practitioners, educators and workplace learning supervisors. Overall, this modified experience of healthcare benefits from considerations of access, equity, collegiality, and how practitioners choose to live, work, learn, stay or go. Change is now the “new normal”!

## 6. Future Directions

There are many potential impacts on pharmacy education, expansions of scope, scope-sharing, professional identity development and activities such as prescribing. In response to the COVID-19 pandemic, actions such as the rapid adoption of online learning, simulation, virtual reality, telehealth counselling and information provision, and AI were arguably taken in haste, but there is now an opportunity to establish the future role within all the learning activities and their contribution to the development of a robust, resilient professional pharmacy profession.

However, of concern, for all the changes made and planned, especially in the rapidly changing times of “Doctor Google” and generative AI, will pharmacy as a profession still exist and still be needed, or might it shatter into other roles? Already, there are medical centre pharmacists, ambulance services pharmacists, prescribing pharmacists, advanced practice pharmacists, and others which are now becoming career tracks rather than role differentiations, such as renal pharmacists and cardiovascular pharmacists by disease or body system, as already exist. We propose that pharmacy will exist and will reinvent itself to serve populations’ needs, informed by its historical pathway and its commitment to the “social contract” with communities as outlined earlier.

Future research can establish this and identify the impact of activities such as those proposed to strengthen capability, adaptability, and resilience, fine-tuning these in readiness for the next challenge. Most importantly, research can establish the appropriate site and access for curation of these materials, not just those such as workplace learning [76], but all the materials in this collaborative global strategy for the future.

## Figures and Tables

**Table 1:** Emerging health support technologies.

Technology	Purpose	Predicted Value
Dispensing robots	Automate medicine distribution, sorting, packaging, counting, and dispensing	US$3.47 billion in 2025 [37]
Personal care robots	Act as socially assistive companions, functional mobility aids, and remote health monitoring units	US$3.1 billion in 2024 [38]
Social contact robots	Provide non-judgmental companionship and emotional support to reduce social anxiety and aid individuals with depression, anxiety, or cognitive disorders; used in schools, therapy clinics, and home settings	US$5.9 billion in 2023 [39]
Health-modified furniture	Designed for healthcare environments with antimicrobial materials, modular features, and monitoring systems to enhance patient safety and caregiver efficiency (e.g., smart beds, modular carts, antimicrobial work surfaces).	US$50.41 billion in 2025 [40]
Mobility aids	Support individuals with limited movement or physical impairments to maintain independence and stability during daily activities.	US$10.4 billion in 2024 [41]
Assistive devices	Improve health and well-being of older adults or those with physical, sensory, or cognitive impairments by enhancing mobility, communication, and daily functioning (e.g., smart canes, AI hearing aids, speech-enabled wheelchairs, home monitoring systems).	US$24.2 billion in 2024 [42]

**Table 2:** Examples of professional development activities included in curricula.

Intervention	Role in Professional Identity Development	Use/Adaptability During COVID
High fidelity simulation including VR	The use of high-fidelity mannequins, virtual reality, or cloud-based dispensing or prescribing software can be considered as alternatives to experiential learning in an online environment [46,47]. These learning modalities can be tailored to fit the needs of learners. For instance, high-fidelity mannequins with injection inserts or simpler early learning tools like oranges have been shown to provide safe opportunities to develop effective professional skills [48]. Konjac models supplemented by video assessment have been reported to develop and maintain microsurgery skills [49]. Whilst pharmacists are not engaged in surgery, konjac models may be a possibility for use in pharmacy education as models for identifying simple injuries such as burns or skin infections.	Simulation may be supported by e-Learning during situations such as COVID-19 or as a learning modality incorporated across campuses or teaching sites such as lecture theatres or hospital wards [50].
Reflective writing	Can serve many purposes at different course levels for health professional students, for example, it may offer students the opportunity to reflect on mistakes of judgement which occurred under supervision, a chance to celebrate successes, identify useful coping strategies, and crucially to examine anxieties that were established to be baseless [51]. Towards the final years, as students’ capabilities develop further, reflective writing and its lessons can be shared for group discussions and peer-to-peer learning once a safe space has been declared and implemented [51]. It is an activity often identified as central to higher education generally and to health professionals’ education as essential in areas of self-esteem, appropriate future focus, concept of professional practice and self-perception of tasks, having a perception [52].	A sustainable and equitable modality, irrespective of resource level (richer or poorer), that enabled students to continue fostering that blending of societal, educational, and personal influences to ensure that professional identity development continued [53].
Interprofessional virtual case studies/debates/vignettes	Interprofessional activities may be situated in a common patient case(s) involving a small number of health profession students to simulate an interprofessional team. These sessions can be held face to face, across campuses and across nations, facilitated by videoconferencing [54]. This learning activity is identified as building collaboration skills, clarifying professional roles and boundaries, and may slightly enhance patient functional outcomes [55].	Although adopted during the COVID-19 pandemic, it had been used before and since, with earlier experiences identifying that prior exposure to interprofessional activities contributes to professional identity development and teamwork [56].

## Data Availability

No new data were created or analysed in this study. Data sharing is not applicable to this article.
